# Supplementary material for: How enhancers regulate wavelike gene expression patterns
Source: eLife. 2023 Jul 11;12:e84969. doi: 10.7554/eLife.84969 (PMC10368423; doi:10.7554/eLife.84969)
Supplement: Supplementary file 1. — frw: forward, rev: reverse. [file elife-84969-supp1.docx]

**Supplementary File 1. List of used primers.** frw: forward, rev: reverse.

| **Description** | **Sequence (5` - 3`)** |
| --- | --- |
| pBPGUw promoter frw + BsaI into NotI | AAATTTGGTCTCCGGCCGCGATCGAGCGCAGCGGTATAAAA |
| pBPGUw promoter rev + BsaI into BamHI | AAATTTGGTCTCGGATCCGTTTGGTATGCGTCTTGTGATTCAAAG |
| pBPGUw promoter-24xMS2-y frw + HindIII | AAATTTAAGCTTGATCGAGCGCAGCGGTATAAAA |
| pBPGUw promoter-24xMS2-y rev + STOP + SbfI | AAATTTCCTGCAGGTTAACCCACAGAATTTGTAGAGACACTAATACTG |
| DSCP frw + HindIII | AAATTTAAGCTTGATAAACGGCCGGCAGCGGTATAAAAGGG |
| DSCP rev + BsaI into BamHI | AAATTTGGTCTCGGATCCGGGCTGCAGATTGTTTAGCTTGTTCAGC |
| hbA frw + BsaI into XmaI | AAATTTGGTCTCCCCGGGCACCCTATTTACGCAACGGCTATTTTC |
| hbA rev + BsaI into HindIII | AAATTTGGTCTCAAGCTTTGGTGGAGATGTTATGGTATGGTCG |
| hbB frw + XmaI | AAATTTCCCGGGCACCCATTGTGACAGCTCGG |
| hbB rev + HindIII | AAATTTAAGCTTTTCTCTGAGGCATAATCCCACTAATTACC |
| runA frw + XmaI | AAATTTCCCGGGGGGTAGTTGTCGGTTAGATGCAATATTGTG |
| runA rev + HindIII | AAATTTAAGCTTCGTTATTCGGAGGTGTCCATTATTGGAAGG |
| runB frw + XmaI | AAATTTCCCGGGCGCAGCGTTATGAAAAATACGGAACAA |
| runB rev + HindIII | AAATTTAAGCTTCCGCCTAGCGAGCATTAGTGC |
| runC frw + BsaI into XmaI | AAATTTGGTCTCCCCGGGGGCTAACATTTGATTCGCACATCGG |
| runC rev + BsaI into HindIII | AAATTTGGTCTCAAGCTTCCTTGTTCAGCAATCACTCAAATATTGTGC |
| KrA frw + BsaI into XmaI | AAATTTGGTCTCCCCGGGAATAATTCCGAAACGCAAAGAGATAGACCC |
| KrA rev + BsaI into HindIII | AAATTTGGTCTCAAGCTTGTGGCATCAAATGGTAGTGACACATCG |
| KrB frw + BsaI into XmaI | AAATTTGGTCTCCCCGGGGTGGCATCAAGTAGTAGTGAGACATCG |
| KrB rev + BsaI into HindIII | AAATTTGGTCTCAAGCTTCTAACGGTGTAGGGTTTGCATAGCAAA |
| KrC frw + BsaI into XmaI | AAATTTGGTCTCCCCGGGTAATCTGAGGAGGCGACGTCTAGC |
| KrC rev + BsaI into HindIII | AAATTTGGTCTCAAGCTTCGATGTCTCACTACTACTTGATGCCAC |
| KrD frw + BsaI into XmaI | AAATTTGGTCTCCCCGGGTTCCATAGCCGACTTGATGCACAACC |
| KrD rev + BsaI into HindIII | AAATTTGGTCTCAAGCTTGCTAGACGTCGCCTCCTCAGATTC |
